# Transmission of *Coxiella burnetii* by ingestion in mice

**DOI:** 10.1017/S0950268820000059

**Published:** 2020-02-05

**Authors:** H. K. Miller, R. A. Priestley, G. J. Kersh

**Affiliations:** Rickettsial Zoonoses Branch, Centers for Disease Control and Prevention, Atlanta, Georgia, USA

**Keywords:** Alimentary tract, Coxiellae, Q fever, virulence, zoonoses

## Abstract

*Coxiella burnetii*, the causative agent of Q fever, is widely present in dairy products around the world. It has been isolated from unpasteurised milk and cheese and can survive for extended periods of time under typical storage conditions for these products. Although consumption of contaminated dairy products has been suggested as a potential route for transmission, it remains controversial. Given the high prevalence of *C. burnetii* in dairy products, we sought to examine the feasibility of transmitting the major sequence types (ST16, ST8 and ST20) of *C. burnetii* circulating in the United States. We delivered three strains of *C. burnetii*, comprising each sequence type, directly into the stomachs of immunocompetent BALB/c mice via oral gavage (OG) and assessed them for clinical symptoms, serological response and bacterial dissemination. We found that mice receiving *C. burnetii* by OG had notable splenomegaly only after infection with ST16. A robust immune response and persistence in the stomach and mesenteric lymph nodes were observed in mice receiving ST16 and ST20 by OG, and dissemination of *C. burnetii* to peripheral tissues was observed in all OG infected mice. These findings support the oral route as a mode of transmission for *C. burnetii*.

## Introduction

*Coxiella burnetii* is a bacterial pathogen that is widely present in dairy products across the United States and many other parts of the world. *C. burnetii* DNA has been detected in bovine, ovine and caprine milk, cheeses, yogurt, creams and butters [1–4]. Viable *C. burnetii* has been isolated from hard cheeses made with unpasteurised milk even after 8 months of ripening [5]. It has been found in 90% of bulk milk tank samples in the United States and unless properly inactivated, viable *C. burnetii* can survive in refrigerated milk up to 42 months [6, 7]. Concentrations of *C. burnetii* in milk can reach as high as 10^6^ cells in one 8 ounce glass [6, 8].

*C. burnetii* is the causative agent of Q fever in humans and coxiellosis in animals [9, 10]. Q fever can present as an acute febrile illness, hepatitis or pneumonia. It is often self-limiting with a low mortality rate. Chronic Q fever can appear months to years after the initial infection and often manifests as endocarditis or vascular infection. Chronic Q fever endocarditis is fatal without treatment and antibiotic treatment is recommended for at least 18 months [10]. Infection is most often attributed to inhalation of aerosolised bacteria shed from infected domestic ruminants [9]. Ingestion as an infectious route for *C. burnetii* has been studied to some degree in goats, dogs, pigeons, rodents and humans [11–22]. Despite this, the role of oral transmission remains controversial. Human studies are confounded by the lack of appropriate controls coupled with unknown pathogenicity and dosage of study strains. To date, no studies in rodents have delivered physiologically relevant dosages of *C. burnetii* directly to the stomachs of immunocompetent animals. Furthermore, virulence amongst different *C. burnetii* strains can vary drastically [23–25], and little is known about the pathogenicity of *C. burnetii* strains currently circulating in dairy products in the United States. As of 2011, the sale of raw milk was legal in 30 states, an increase from 22 in 2004 [26]. In 2011, an outbreak of Q fever occurred in Michigan, believed to be the result of consuming raw milk [17]. Viable *C. burnetii* has been isolated from multiple samples of unpasteurised milk across many states [4, 27].

Three major sequence types (STs) of *C. burnetii* have recently been identified in the United States by single-nucleotide polymorphism-based analyses [4, 28]. ST8 is associated with goats and is responsible for multiple outbreaks of Q fever [28]. ST20, linked to dairy cattle, is widespread in dairy products, while ST16/26s are not closely associated with any one species [4, 28]. Prior to 2007, no ST20 isolates had been collected in the United States; therefore, little is known about the pathology of these new strains. Given the close species association of *C. burnetii* strains coupled with the rising popularity of goats and their milk-based products in the United States, we sought to examine whether consumption as a route of infection is possible or strain dependent amongst these relevant sequence types.

## Methods

### Ethical statement

The authors assert that all procedures contributing to this work comply with the ethical standards of the relevant national and institutional guides on the care and use of laboratory animals. All animal experiments were performed according to an animal protocol approved by the CDC Institutional Animal Care and Use Committee.

### Bacterial strains and growth conditions

The *C. burnetii* strains used in this study include Nine Mile (NM) phase I (RSA493), an ST16 isolated from a tick in 1935 [29], CM-SC1, an ST20 strain, isolated in 2007 from unpasteurised cow's milk purchased in South Carolina [4, 27] and GP-CO1, an ST8 strain, isolated in 2007 from the placenta of a goat associated with a human Q fever outbreak located in Colorado [30]. Strains were cultured in RK-13 cells and purified by digitonin lysis [31]. Stocks were kept frozen at −80 °C in sucrose phosphate glutamate buffer until use.

### Mouse infections

Male BALB/c mice, 4–6 weeks of age, were purchased from Charles River Laboratories. This mouse strain is considered intermediately sensitive to *C. burnetii* infection [32, 33]. Mice were housed in a Tecniplast Isocage system (Tecniplast, Exton, PA) in an ABSL3 facility and given food and water *ad libitum*. Mice were orogastrically inoculated using a feeding needle to deliver a target inoculum of 1 × 10^6^ genome equivalents (GE) (actual GE administered: 1.24 × 10^6^ of NM, 2.56 × 10^6^ of CM-SC1 and 3.05 × 10^6^ of GP-CO1) in a 100 µl volume of phosphate-buffered saline (PBS). The dosing volume represents 4–5 ml per kg of body weight and was selected to reduce the risk of gavage-related reflux. No animals receiving inoculums via oral gavage (OG) displayed any signs of respiratory distress that would suggest aspiration of dosing material. Mice receiving the same dose of *C. burnetii* in 100 µl of PBS by intraperitoneal injection (IP) served as positive controls. Negative control mice were given 100 µl of sterile PBS by OG inoculation. The OG-infected, IP-infected and uninfected mice were maintained in separate HEPA-filtered isolator cages. Experiments with the three *C. burnetii* isolates were carried out independently with four OG-infected mice, two IP-infected mice and two PBS OG mice for each of three time points. Mice were monitored for clinical symptoms and weighed periodically for 21 days post-infection. On days 7, 14 and 21, mice were euthanised and blood was collected by cardiac puncture. Lungs, spleen, liver, stomach, small intestine, caecum and colon were aseptically removed. Spleens were weighed before further processing. Tissues were homogenised in PBS using 3.0 mm triple-pure zirconium beads in a BeadBug 6 microtube homogeniser (Southern Labware, Cumming, GA) with a speed of 7.0 m/s for five 30 s cycles with a 30 s pause between each cycle.

### Quantitative polymerase chain reaction (PCR)

To quantify *C. burnetii* DNA in blood and tissues, total genomic DNA was extracted from 200 µl of blood or homogenised tissues using the QIAamp DNA mini kit (Qiagen, Valencia, CA), according to the manufacturer's instructions. Gut tissues (small intestine, caecum and colon) were processed using the QIAamp Fast DNA Stool mini kit (Qiagen, Valencia, CA), according to the manufacturer's instructions with the following modifications. A 200 µl volume of homogenised tissue was added at a 1:1 ratio with the InhibitEX buffer. Quantitative PCR was performed using primers specific to *com1*, as previously described [34]. Cycle threshold (C_t_) data for *C. burnetii com1* were normalised to murine Actb (Applied Biosystems, Waltham, MA) and normalised cycle threshold values (ΔC_t_) were transformed using 2^−ΔCt^/10^−6^ [35], and reported as arbitrary quantity units.

### Serology

Serum samples were screened for both phase I and phase II anti-*C. burnetii* immunoglobulin G (IgG) by indirect immunofluorescence antibody test (IFA) as previously described [36]. Blood was separated in a 1.1 ml microtube containing serum gel with a clotting activator (Starstedt, Nϋmbrecht, Germany) at 10 000 ***g*** for 5 min. Geometric mean titre (GMT) was calculated as described previously [37].

### Statistical analysis

Statistical significance of weight loss and splenomegaly data was determined by Student's *t*-test. Data were analysed using GraphPad Prism 7.01 software (GraphPad). For all analysis, *P* < 0.05 was deemed significant.

## Results

### The NM strain effects weight gain and splenomegaly following oral infection in immunocompetent BALB/c mice

**Fig. 1.**
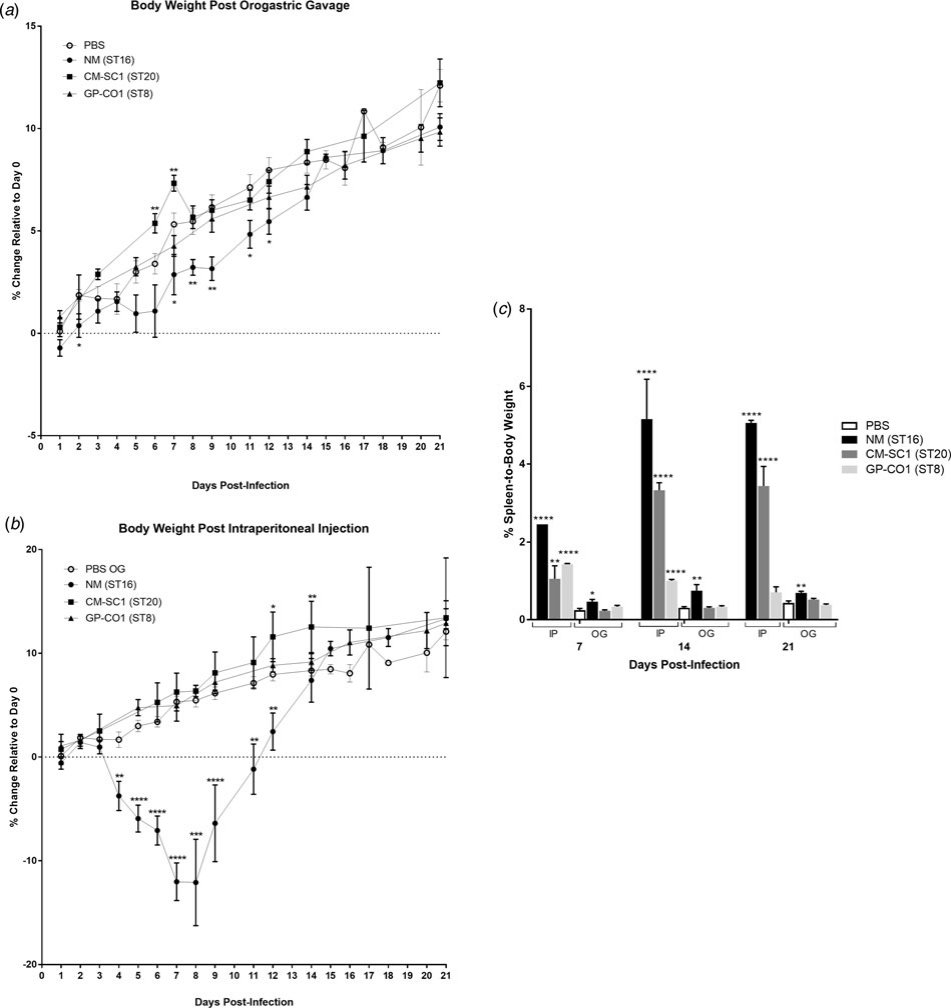
Body weight and splenomegaly following oral infection with *C. burnetii* in immunocompetent BALB/c mice. Mice were infected with 10^6^ of either NM (•), CM-SC1 (■) or GP-CO1 (▲) via (a) OG or (b) IP. Sterile PBS (○) was delivered via OG as negative controls. Data are presented as the mean percentage change in weight relative to day 0 ± S.E.M. (c) Spleen-to-body weight (mean ± S.E.M.) is shown for mice infected with NM (black), CM-SC1 (dark grey), GP-CO1 (light grey) or PBS (white) via OG or IP. *P < 0.05, **P < 0.01, ***P < 0.001 and ****P < 0.0001 as determined by Student's *t*-test relative to PBS OG negative controls.

We delivered three different strains of *C. burnetii*, Nine Mile (NM), CM-SC1 and GP-CO1 directly into the stomachs of BALB/c mice via OG and noted no signs of illness through 21 days post-infection (pi). Among mice given the same inoculums via IP injection, only NM-infected mice displayed ruffled fur from day 4 through day 12. None of the mice died prior to being euthanised. None of the mice infected with CM-SC1 or GP-CO1 displayed decreased weight relative to PBS controls regardless of the administration route (Fig. 1a and b). By day 8 pi, NM IP mice had lost an average of 12.09% while PBS OG control mice had gained 5.47% (*P* < 0.0001). Although, NM OG mice displayed no weight loss relative to day 0, weight gain was decreased 1.9-fold (*P* < 0.01) relative to PBS OG mice by day 9 pi.

Splenomegaly was observed in all three strains when delivered via IP albeit with variable timing and intensity (Fig. 1c). The largest increase in percent spleen-to-body weight occurred in NM IP-infected mice across all time points studied, with a 17-fold increase (*P* < 0.0001) relative to PBS at day 14 pi. Splenomegaly increased across the study period for CM-SC1 IP-infected mice, with an eightfold increase (*P* < 0.0001) by day 21 pi. The GP-CO1 IP cohort had a sixfold increase (*P* < 0.0001) at day 7 pi, which fell to 3.3-fold (*P* < 0.0001) by day 14 pi and was no longer significant relative to PBS OG controls by day 21 pi. Among the mice infected via OG, only NM was capable of inducing significant spleen enlargement with 1.9-fold (*P* < 0.05), 2.5-fold (*P* < 0.01) and 1.6-fold (*P* < 0.01) increases relative to PBS OG for days 7, 14 and 21 pi respectively.

### Mice mount a robust immune response against NM and CM-SC1 strains of *C. burnetii* delivered directly into the stomach

**Fig. 2.**
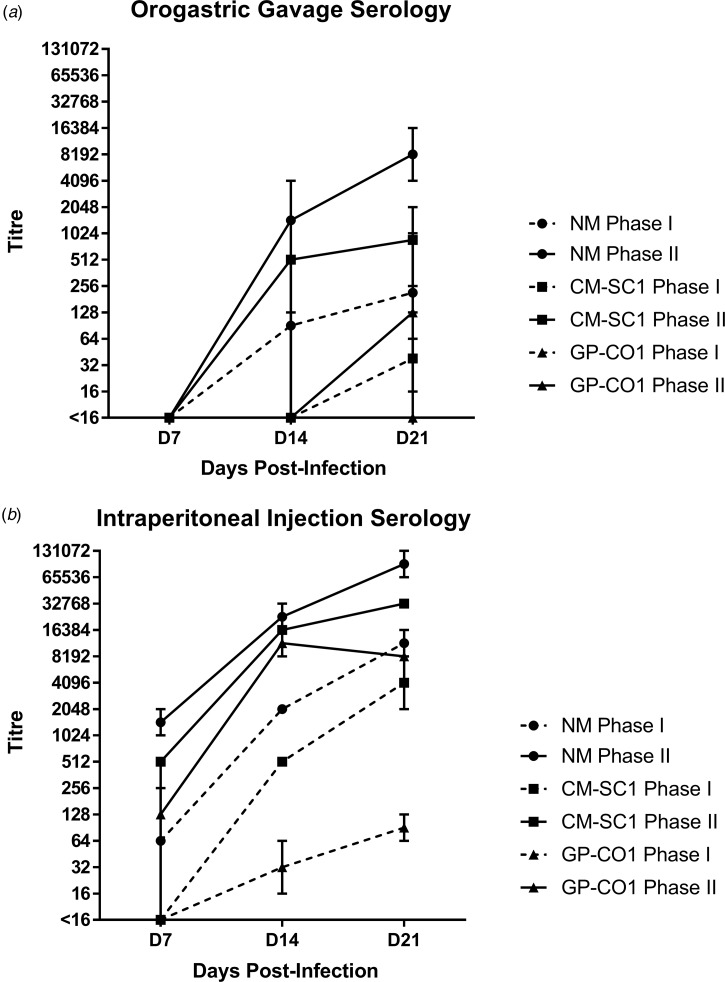
Serological response following oral infection with *C. burnetii* in immunocompetent BALB/c mice. Serum was analysed for the presence of IgG antibodies against phase I (dashed line) and phase II (solid line) *C. burnetii* by indirect IFA. The GMT ± range is shown for mice receiving either NM (•), CM-SC1 (■) or GP-CO1 (▲) via (a) OG or (b) IP. Mice receiving PBS were seronegative at every time point (data not shown). Values below the limit of detection are indicated as <16.

Sera of mice were analysed for the presence of antibodies against *C. burnetii* phase I (PhI) and phase II (PhII) antigens using IFA. Sera from PBS-treated mice were negative against both antigens at all time points (data not shown). Mice receiving NM orally had no detectible antibodies at day 7 pi; however, all mice seroconverted by day 21 pi with a GMT of 215 (range: 128–1024) for PhI and 8192 (range: 4096–16 384) against PhII (Fig. 2a). Only one of the mice receiving CM-SC1 by OG seroconverted by day 14 pi; however, by day 21 pi all had antibody titres against *C. burnetii*, with a GMT of 38 (range: 16–64) for PhI and 861 (range: 256–2048) for PhII. Among mice infected with GP-CO1 by OG, only one seroconverted by day 21 pi with a titre of <16 for PhI and 128 for PhII.

The highest antibody titres were observed for mice receiving NM via IP injection with a GMT of 11 585 (range: 8192–16 384) for PhI and 92 682 (range: 65 536–131 072) for PhII by day 21 pi (Fig. 2b). The GMT for CM-SC1 was 4096 (range: 2048–8192) for PhI and 32 768 (range: 32 786–32 768) for PhII by day 21 pi. The lowest titres among IP-injected mice were observed for those receiving GP-CO1 with a GMT of 91 (range: 64–128) for PhI and 8192 (range: 8192–8192) for PhII by day 21 pi.

### *Coxiella burnetii* NM, CM-SC1 and GP-CO1 strains are able to escape the gastrointestinal tract and disseminate to peripheral tissues

**Fig. 3.**
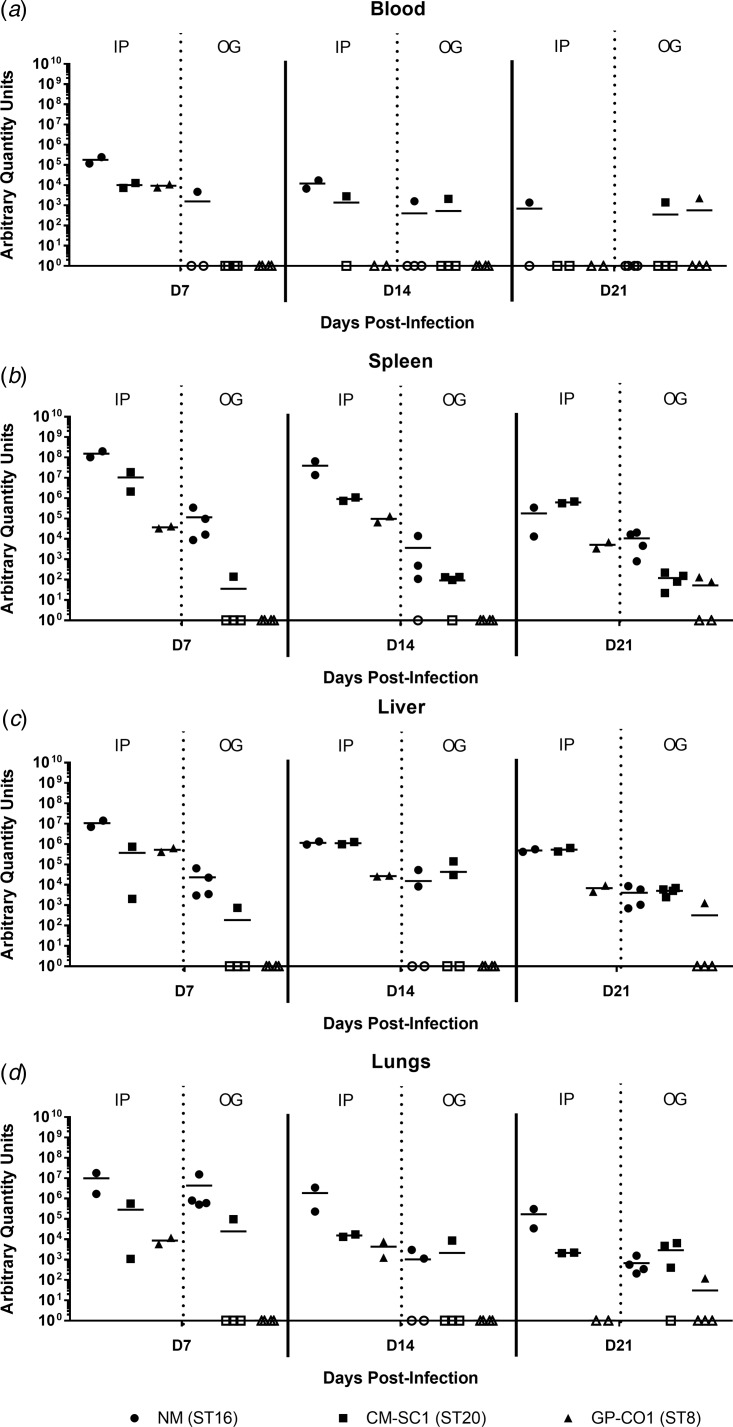
Dissemination following oral infection with *C. burnetii* in immunocompetent BALB/c mice. (a) Blood, (b) spleens, (c) livers and (d) lungs were analysed for the presence of *C. burnetii* DNA by quantitative PCR. Cycle threshold (C_t_) data for the *C. burnetii com1* gene were normalised to murine Actb (Applied Biosystems, Waltham, MA) and normalised cycle threshold values (ΔC_t_) were transformed using 2^−ΔCt^/10^−6^ [35], and reported as arbitrary quantity units. Individual data points and the mean (bar) are shown for mice receiving either NM (•), CM-SC1 (■) or GP-CO1 (▲) via OG or IP. Open symbols represent values below the limit of detection. No *C. burnetii* DNA was detected in any PBS control mice (data not shown).

Murine blood and tissues were analysed for the presence of *C. burnetii* DNA using quantitative PCR. Tissues from PBS-treated mice were negative at all time points (data not shown). All mice infected by IP injection had bacterial DNA in the blood at day 7, which decreased throughout the 21-day study period (Fig. 3a). Amongst the cohort of mice infected by OG, no more than one mouse was positive for Coxiella in the blood at any time point tested, regardless of the infecting strain. On days 7 and 14 pi, splenic levels of *C. burnetii* DNA were highest in NM followed by CM-SC1 and GP-CO1 regardless of the administration route (Fig. 3b). Among IP-infected mice, NM was 15-fold higher and 4000-fold higher by day 7 than CM-SC1 and GP-CO1 infected mice, respectively. For mice infected by OG, splenic bacterial loads were highest for NM at day 7 followed by a 32-fold decrease by day 14. Subsequent to CM-SC1 and GP-CO1 OG infection, the number of positive mice peaked at day 21 with four and two mice, respectively. Similar trends were observed in the liver and lungs for both IP- and OG-infected mice (Fig. 3c and d). Regardless of the infecting strain or the tissue type, mice infected by IP had higher bacterial loads relative to OG-infected mice.

### *Coxiella burnetii* NM and CM-SC1 strains are able to persist in the stomach and mesenteric lymph nodes following oral gavage

**Fig. 4.**
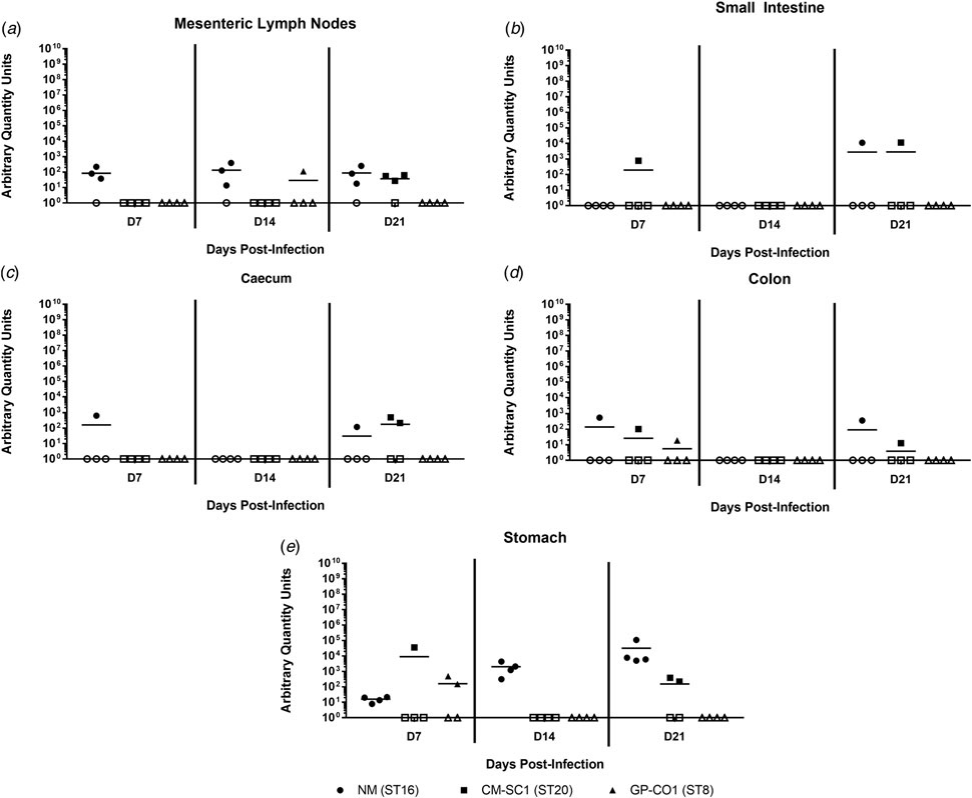
Colonisation of the gastrointestinal tract following oral infection with *C. burnetii* in immunocompetent BALB/c mice. (a) Mesenteric lymph nodes (MLN), (b) small Intestines, (c) caecum, (d) colon and (e) stomach were analysed for the presence of *C. burnetii* DNA by quantitative PCR. Cycle threshold (C_t_) data for the *C. burnetii com1* gene were normalised to murine Actb (Applied Biosystems, Waltham, MA) and normalised cycle threshold values (ΔC_t_) were transformed using 2^−ΔCt^/10^−6^ [35], and reported as arbitrary quantity units. Individual data points and the mean (bar) are shown for mice receiving either NM (•), CM-SC1 (■) or GP-CO1 (▲) via OG. Open symbols represent values below the limit of detection. No *C. burnetii* DNA was detected in any PBS control mice (data not shown).

To date, no data are available as to whether *C. burnetii* can persist in the gastrointestinal tract following oral entry. To address this, we analysed tissues of the alimentary system subsequent to OG with NM, CM-SC1 or GP-CO1. Regardless of the infecting strain, *C. burnetii* DNA was only transiently identified in the small intestine, caecum and colon throughout 21 days pi (Fig. 4a–c). At each time point tested, NM was detected in the mesenteric lymph nodes (MLN) of three of the mice (Fig. 4d). By day 21, MLN of three mice were positive following infection with CM-SC1, while only one GP-CO1 OG mouse had positive MLNs at day 14. As demonstrated in Figure 4e, GP-CO1 and CM-SC1 were identified in stomachs of two of the mice by day 7 and day 21, respectively. Interestingly, NM was detected in the stomachs of all OG mice at every time point and mean quantity increased 2000-fold from day 7 to 21. Gastrointestinal tract tissues from IP-treated mice were positive at all time points, except for the caeca of GP-CO1-infected mice which were positive through day 14 pi (data not shown).

## Discussion

We analysed the ability of three strains of *C. burnetii* to infect immunocompetent BALB/c mice when introduced directly into the stomach. The three strains, Nine Mile (ST16), CM-SC1 (ST20) and GP-CO1 (ST8) comprise the three major sequence types of *C. burnetii* currently circulating in the United States [4, 28]. All three strains led to infection in mice following OG, evidenced by the ability to detect *C. burnetii* DNA in tissues coupled with an antibody response (IFA; PhII titre of >32). These findings indicate that the oral route is a viable route of transmission for these strains. Each strain was also injected into the peritoneal cavity of mice at the same dose, which resulted in more severe disease in all three strains. These findings demonstrate that only a portion of the Coxiella are able to transition from the gastrointestinal tract and disseminate. Further studies are needed to determine whether this decrease in pathology relative to IP is dose dependent and how OG relates to aerosol challenge.

We demonstrate herein that *C. burnetii* is present in the stomachs of mice 21 days after oral entry and that the level of gastric NM increased dramatically over the study period. These findings suggest that *C. burnetii* is able to avoid elimination by peristalsis, invade the gastric epithelium and replicate; however, the mechanism by which this occurs remains to be determined. Furthermore, it is unclear whether Coxiella localises to specific regions within the stomach and how long it is able to persist. These findings raise concerns regarding repeated exposure through continued consumption of raw milk, which may support colonisation of the stomach. Similar to other enteropathogenic bacteria such as *Salmonella enterica* serovar Typhimurium, *Yersinia enterocolitica* and *Yersinia pseudotuberculosis*, *C. burnetii* is capable of colonising the MLNs in orally infected mice, suggesting that Coxiella is able to penetrate the intestinal epithelial barrier [38–40]. Work with the NM strain in bovine intestinal epithelial cells *in vitro* showed enhanced susceptibility to invasion by *C. burnetii* relative to lung and placental epithelial cell lines; however, replication within the intestinal cells was limited [41]. The mechanism by which Coxiella escapes the gastrointestinal tract is not understood.

We observed varying degrees of virulence amongst the three strains tested; however, this was independent of the administration route. Infection with the well-characterised laboratory strain, Nine Mile, resulted in more severe disease based on all aspects examined. This strain was originally isolated from a tick in 1935 and has since been passaged in guinea pigs 307 times [29]. It is possible that these methods have led to adaptation of this strain to rodents, which may account for the increased pathogenicity relative to CM-SC1 and GP-CO1, which have only been passaged one to two times in mice. CM-SC1 belongs to the sequence type 20, which is widespread in cattle across the United States, while GP-CO1 belongs to the goat-associated ST8s. Despite the ST8s being frequently linked to Q fever outbreaks in the United States, GP-CO1 was the least virulent of the strains tested. It is possible that ST8 strains are less likely to induce a robust immune response, thereby allowing for long-term persistence in the host. This is supported by the drastically reduced phase I specific antibody titres observed in GP-CO1 infected mice relative to CM-SC1, despite having comparable bacterial loads in the blood (Fig. 1a and 2). In the absence of additional test strains within each sequence type, it is not possible to demonstrate conclusively that virulence is distinct among these groups. However, these strains show remarkably consistent species association and *in vitro* growth characteristics, which supports the notion that they may have similar characteristics *in vivo* as well [4, 28]. Furthermore, similar patterns of virulence were observed in a study utilising a guinea pig IP model, where NM and another ST16, Ohio, were determined to be the most virulent, followed by Q195, an ST20, and finally the ST8s, Q238 and Priscilla [23]. Further studies are needed to determine whether these findings are representative of each sequence type and whether the pathogenicity observed herein is consistent following aerosol challenge.

## Disclaimer

The findings and conclusions in this report are those of the authors and do not necessarily represent the views of the CDC.
